# Correspondence

**Published:** 1877-09

**Authors:** W. S. Caldwell

**Affiliations:** Warren, Ill.; Edinburgh


### Correspondence.

LETTER FROM EDINBURGH—MR. LISTER AND HIS ANTISEPTIC METHOD—HIS REMOVAL FROM EDINBURGH TO LONDON, AND ACCEPTANCE OF A POSITION IN KING'S COLLEGE.

*To the Editor of the Chicago Med. Journal and Examiner:*

Whatever may be the opinion of the profession at large, as to the merits of Mr. Lister's ideas on the subject of his antiseptic treatment in surgery, he has certainly achieved an enviable reputation at home.

Even the emperor, Dom Pedro, during his flying visit to this city, paid him the compliment of calling at the infirmary to witness his antiseptic dressings, as applied in the wards.

As your readers are probably aware, Mr. Lister has lately accepted a position at King's College, London, and will sever his connection with the University of Edinburgh at the close of the present term, and will then move to London, and make that his permanent residence.

As the school to which he goes is much smaller than the one here, and the number of beds of which he will have charge fewer than those he here controls, some surprise has been expressed by his friends at his accepting his new position.

His reply is that he hopes in London to be able to diffuse more widely his views on the germ theory of the contamination of open wounds after surgical operations.

Though his practice here is a very lucrative one, having inherited a large fortune from his father, he is able to leave out of the question the matter of money in his contemplated move.

He claims that the reason why such varied results are obtained by those who attempt to practice antiseptic surgery, is that some are not sufficiently imbued with its importance to carry out its teachings in all their minutiæ, and that a certain other number attempt its practice without being sufficiently well informed on the subject to be able to operate as he directs.

As an illustration of the latter class, he mentioned to me the name of a New York professor who only remained in Edinburgh a single day, imagined that he comprehended the whole subject, but when he returned home and began its practice, he failed to obtain satisfactory results, and raised the cry of humbug; when Mr. Lister says positively that he knows from the character of some of his criticisms that he never understood the subject sufficiently well to practice it intelligently.

To illustrate the extreme care that is necessary in the practice of antiseptic surgery, he related to me the following case. He was opening under the spray a psoas abscess, the result of disease of the spine. His assistant who was working the spray apparatus, becoming annoyed at a student behind him, and kicking at him, allowed the jet of spray for just an instant to be so directed as not to cover the opening he had just made. In great anger the doctor told his assistant that if that patient died he might consider himself the cause of her death.

She did die, and Mr. Lister believes that the misdirection of that jet of spray for the small fraction of a minute was the cause of her death.

It is in this last mentioned disease that Mr. Lister claims the most eminent success for his treatment.

That it is the contamination of the diseased parts by septic germs that gain access to the diseased surface after the ordinary modes of opening the abscess, or allowing them to discharge themselves, is proven by the increased gravity of the symptoms that almost uniformly follow such discharge.

He opens these abscesses antiseptically as soon as possible, cutting down deeply, if necessary, puts on his antiseptic dressings, always changing them under the spray; and keeps his patient quietly in bed until the parts heal, which he claims they very uniformily do. He puts in a Chassaignac's drainage tube, which he allows to remain during the whole treatment, cutting it shorter as the parts heal from the bottom.

To show some of the results of antiseptic surgery as done by Mr. Lister, I have taken the following notes from cases in the wards of the infirmary, which cases I visited every day, and recorded the temperature myself.

John A., aged 23, amputation of the femur at the union of the lower and middle third for malignant disease of the knee joint. Highest temperature, $99\frac{4}{10}°$ Far.; never but little acceleration of pulse.

William G., aged 14; excision of knee joint for anchylosis, accompanied by extreme flexion. This case was a very difficult one, indeed, requiring tenotomy, and the removal of a large section of the femur. Highest temperature, $100\frac{3}{10}°$ Far.

Next case was that of William G., aged 34. Amputation at the hip joint for a chondroma of the upper portion of the femur. Highest temperature, $99\frac{4}{10}°$ Far.

The patient was perfectly comfortable after twenty-four hours, and went on to convalescence without an untoward symptom.

Mr. Lister insists that all surgical operations, except in rare cases, should be performed in the most slow and deliberate manner. He says that Mr. Liston introduced the dashing mode of operating into this country, much to the detriment, as he thinks, of the patients he operated on. In the case detailed above of excision of the knee joint, he was one hour and a half in performing the operation.

W. S. Caldwell, M. D., of Warren, Ill.

Edinburgh, July, 1877.

